# Individual Differences in Anchoring Effect: Evidence for the Role of Insufficient Adjustment

**DOI:** 10.5964/ejop.v15i1.1691

**Published:** 2019-02-28

**Authors:** Predrag Teovanović

**Affiliations:** a Faculty for Special Education and Rehabilitation, University of Belgrade, Belgrade, Serbia; University of Belgrade, Belgrade, Serbia

**Keywords:** anchoring effect, individual differences, dual-process theories, intelligence, cognitive reflection

## Abstract

Although the anchoring effect is one of the most reliable results of experimental psychology, researchers have only recently begun to examine the role of individual differences in susceptibility to this cognitive bias. Yet, first correlational studies yielded inconsistent results, failing to identify any predictors that have a systematic effect on anchored decisions. The present research seeks to remedy methodological shortcomings of foregoing research by employing modified within-subject anchoring procedure. Results confirmed the robustness of phenomenon in extended paradigm and replicated previous findings on anchor’s direction and distance as significant experimental factors of the anchoring effect size. Obtained measures of individual differences in susceptibility to anchoring were fairly reliable but shared only small portion of variability with intelligence, cognitive reflection, and basic personality traits. However, in a group of more reflective subjects, substantial negative correlation between intelligence and anchoring was detected. This finding indicates that, at least for some subjects, effortful cognitive process of adjustment plays role in the emergence of the anchoring effect, which is in line with expectations of dual-process theories of human reasoning.

Anchoring effect refers to a systematic influence of initially presented numerical values on subsequent judgments of uncertain quantities, even when presented numbers are obviously arbitrary and therefore unambiguously irrelevant. A simple two-step procedure, usually referred to as a standard paradigm of anchoring, was initially introduced in the seminal work of Tversky and Kahneman (1974). First, people were instructed to make a judgment if some quantity, such as the percentage of African countries in the UN, is higher or lower than value randomly generated by spinning a wheel of fortune in their presence. Subjects were then asked to provide their own numerical estimates for the very same quantity. Results showed notable effect of arbitrary values on participants’ estimates (e.g. percentage of African countries in the UN was estimated on 25 and 45 for groups that were presented with anchors 10 and 65, respectively).

Exceptional easiness of experimental elicitation of the anchoring effect was demonstrated in various domains, in both laboratory and real-world settings, and for both novice and expert subjects (for reviews of literature, see Bokulić & Polšek, 2010; Furnham & Boo, 2011). Anchoring effect is so pervasive that even highly implausible anchors (e.g. “Did Gandhi lived to be 140?”) affect judgments. However, it seems that relation between anchor distance and the size of the anchoring effect is curvilinear - moderate anchors lead to stronger effect in comparison extreme ones (Mussweiler & Strack, 2001; Wegener, Petty, Detweiler-Bedell, & Jarvis, 2001). Besides, the larger effect was observed for anchors set above, in comparison to anchors set below correct answer (Hardt & Pohl, 2003; Jacowitz & Kahneman, 1995; Jasper & Christman, 2005).

According to Tversky and Kahneman (1974), anchoring effect is the product of anchoring and adjustment heuristic. Estimates are made starting from anchor value which is then adjusted in a deliberate fashion, step by step until the satisfactory answer is reached. A number of steps is often insufficient since people stop adjusting as soon as they reach the first answer that seems acceptable. Anchoring is seen as a consequence of this prematurely ended serial process.

An alternative explanation of anchoring assumes that anchor automatically cues positive test strategy, i.e. intuitive search for pieces of evidence that confirm anchor as a possible true answer for target quantity. Information that is consistent with the anchor is more easily accessible on successive estimation task, which in turn affects final estimates (Chapman & Johnson, 1999; Mussweiler & Strack, 1999; Strack & Mussweiler, 1997).

Recently, several attempts were made to integrate two competing accounts of anchoring (see e.g. Epley, 2004; Epley & Gilovich, 2005, 2006; Kahneman, 2011; Simmons, LeBoeuf, & Nelson, 2010). Endorsing dual-process theories of human reasoning, Kahneman (2011) noted that anchoring results from both automatic activations of anchor-consistent knowledge and re-adjustments from anchor as distinct cognitive processes – the first, fast and intuitive (Type 1), and the second, more demanding, slow and effortful (Type 2). Latter requires controlled attention, and relies on working memory capacities. It has been shown that adjustment decreases when cognitive resources are depleted either by concurrent cognitive task or alcohol consumption (Epley & Gilovich, 2006), and increases when instructions aimed to enhance mental efforts are introduced (Adame, 2016; Epley & Gilovich, 2005; Mussweiler, Strack, & Pfeiffer, 2000; Simmons et al., 2010).

Additional findings regarding the role of Type 2 processing in the emergence of the anchoring effect are needed. Since anchors may not affect all people equally, one of promising lines of research comes from the individual differences perspective. Starting from the assumptions of dual-process theories which state that people reliably vary in capacities to successfully perform Type 2 processes, and that these differences highly correspond to individual differences in intelligence (Evans & Stanovich, 2013; Stanovich & West, 2000, 2008), one could expect to observe negative correlation between anchoring and intelligence. However, recent studies failed to reveal such association (Furnham, Boo, & McClelland, 2012; Jasper & Ortner, 2014; Stanovich & West, 2008; Welsh, Delfabbro, Burns, & Begg, 2014; for minor exceptions, see Bergman, Ellingsen, Johannesson, & Svensson, 2010; Jasper & Christman, 2005).

Apart from the ability to successfully perform them, individuals also vary in mere readiness to undertake Type 2 processes at all (see e.g. Evans & Stanovich, 2013; Stanovich, 2009). Individual differences in this propensity are usually assessed by cognitive reflection test (CRT; Frederick, 2005), devised with the intention to differentiate between impulsive subjects who are prone to report response that first comes to mind (Type 1), and reflective subjects, who are able to resist to the first response and try to provide more thoughtful (Type 2) answer. No direct effect of cognitive reflection on anchored decision was observed in previous studies (Bergman et al., 2010; Jasper & Ortner, 2014; Oechssler, Roider, & Schmitz, 2009; Welsh et al., 2014). However, following the assumptions of dual process theories, it seems plausible to hypothesize that effects of intelligence and cognitive reflection on anchoring effects might be in interaction. More precisely, one could expect that individual differences in intelligence play role in the emergence of the anchoring effect only when Type 2 processes are initiated, i.e. when subjects’ reflectivity is high. On the other side, intelligence and anchoring should not be related if subjects are more impulsive, i.e. prone to rely on intuitive Type 1 processes.

Finally, it seemed practically worthwhile to empirically examine correlations that anchoring might have with personality traits. Previous studies on relation between anchoring effect and Big Five personality traits have brought mixed results. For example, McElroy and Dowd (2007) detected a modest positive correlation between susceptibility to anchoring and openness to experience, while Eroglu and Croxton (2010) failed to replicate this finding, but encountered on significant, although relatively small, associations with agreeableness, conscientiousness, and introversion. On the other side, Caputo (2014) reports that anchoring was negatively related to agreeableness and openness. Other studies reported on sporadic and practically negligible correlations between Big Five personality traits and susceptibility to anchoring (Furnham et al., 2012; Jasper & Ortner, 2014).

## Research Aims and Hypotheses

From the experimental perspective, research was aimed to examine how both direction and relative distance of anchor contribute to the anchoring effect size. A non-linear relationship between anchor distance and the size of the anchoring effect (Mussweiler & Strack, 2001; Wegener et al., 2001), as well as a stronger effect of positively directed anchors (Hardt & Pohl, 2003; Jacowitz & Kahneman, 1995; Jasper & Christman, 2005), were expected. In order to ensure that relative distance of the anchor is closely the same for all participants, the standard paradigm was extended by introducing pretest session (see Method section). This procedural intervention also allowed to examine if reliable individual differences in susceptibility to the anchoring effect could be collected.

The main correlational aim of the research was to determine whether traditional psychometric constructs can predict anchoring effect, with an expectation that relation between anchoring and intelligence is moderated by cognitive reflection (Evans & Stanovich, 2013; Kahneman, 2011; Stanovich, 2009). On the other side, considering the inconsistent findings of previous studies, it was hard to come out with strong expectations regarding relation between anchoring effect and Big Five personality traits.

## Method

### Participants

A total of 236 special-education undergraduate students (214 females; age *M* = 19.83, *SD* = 1.31) participated in this study that was part of a wider research on cognitive biases (see Teovanović, 2013; Teovanović, Knežević, & Stankov, 2015) in return for partial course credit. Participants gave their informed consent before taking part in the study.

### Anchoring Experiment

#### Material

A set of 24 general knowledge questions, all required numerical answers, was used in the anchoring experiment. Relatively difficult questions, covering various topics, were chosen with the intention to induce conditions of uncertainty. Participants were not expected to know the precise answers but to provide approximate estimates of target quantities.

#### Design

The anchoring experiment employed 2 x 4 within-subject design, with anchor direction and anchor distance as repeated factors. Anchor direction had two levels: positive, for anchors placed above, and negative, for anchors placed below the values of initial estimates. Four levels of relative anchor distance were set by adding or subtracting the particular portion (20%, 40%, 60%, or 80%) of the initial estimate’s value. Three different general knowledge questions were used in each of eight experimental conditions (see the first two rows in Table 1), resulting in a total of 24 questions.

#### Procedure

Two-session anchoring experiment was administered via a computer program which was used for presentation of instructions and questions, recording of answers, and calculation of individual anchor values. In the first session, questions were presented in a randomized order, one at a time and the participants were instructed to state the answers by using a numeric keypad. Answers were recorded as initial estimates (E1) and used in the subsequent session as the basis for the setting anchors (A) on various levels of distance and direction factors. As previously stated, for each participant (p) on each presented question (q), individual anchor values (A_pq_) were calculated by multiplying initial estimates (E1_pq_) with predetermined values (which ranged between 0.2 and 1.8 across questions) and rounding decimals. At the beginning of the second session, which followed immediately, an instruction was presented on the screen: “In the previous session, you answered a set of questions. In the following one, you will be asked to answer the same questions again. You are allowed, but not obliged, to change your mind and provide amended answers”. Same 24 questions were presented in a new random order, but the standard paradigm of anchoring was applied, i.e. for each question participants were administered a comparative task and a final estimation task. More precisely, the participant was first required to indicate if her/his final response was higher or lower than the value of the specific anchor (A_pq_). After that, a participant would state the final response (E2_pq_) by using the numeric keypad.

#### Measure

Measures of the anchoring effect (AE) were calculated for each participant on each question by using the following formula: AE_pq_ = (E2_pq_ - E1_pq_) / E1_pq_ * 100.

As an index of relative amendment in estimation after introducing of anchor, AE tells about the difference between initial and final estimate in the units of percentage of initial estimate’s value^i^. Since relative anchor distance was also expressed in terms of percentage of the initial estimate, this enabled their direct comparison. For all questions, zero value of AE indicated an absence of difference between the initial and final estimate, i.e. lack of anchoring effect. Maximum magnitude of anchoring effect was determined by the relative anchor distance on specific question (for negative anchors, it had negative values: -20%, -40%, -60%, -80%; for positive anchors, it had positive values: 20%, 40%, 60%, 80%).

#### Data Trimming

In order to control for the unwanted effect of outlying AE values, trimming procedure was applied. The absence of the anchoring effect and the maximum of the anchoring effect determined lower and upper bound of an acceptable range for AE measures. However, two type of data departures were both expected and observed. The first concerns under-anchoring, and it had been registered when AE was lower than zero for positive anchors, i.e. higher than zero for negative anchors. Similarly, over-anchoring was indicated when values of final estimates were smaller than values of negatively directed anchors, i.e. higher than values of positively directed anchors. Measures that had been outside of acceptable range were fenced to meet its boundaries. Corresponding second estimations (E2) were also amended such that applied formula results in trimmed AE measure. A described procedure was applied on 17.5% of all AE and E2 measures (see the last two rows of Table A1 in the Appendix)^ii^.

### Individual Differences Measures

One week prior to the anchoring experiment, several cognitive ability tests and one personality questionnaire were computer administered to the same group of participants. Personal identification numbers were used for matching participants’ data.

#### Raven’s Matrices

Raven’s Matrices (RM; Raven, Court, & Raven, 1979). For each of 18 tasks, participants were asked to identify the missing symbol that logically completes the 3x3 matrix by choosing from among five options. Participants were allowed six minutes to complete the test. Previous studies that had used this instrument reported about its good metric properties (see, e.g., Pallier et al., 2002; Teovanović et al., 2015).

#### Vocabulary Test

Vocabulary Test (Knežević & Opačić, 2011) consists of 56 items of increasing difficulty. Subjects were required to define the words (e.g. “vignette”, “credo”, “isle”) by choosing the answer from among six options. No time limit for the completion of this test was imposed. On average, the participants completed this test in 13.11 minutes (*SD* = 2.09).

#### Cognitive Reflection Test

Cognitive Reflection Test (CRT) was devised as a measure of “the ability or disposition to resist reporting the response that first comes to mind” (Frederick, 2005, p. 36). It consists of only three questions, each triggering most participants to answer immediately and incorrectly. As previously noted, CRT was used to capture individual differences in propensity to engage Type 2 processing.

#### NEO-FFI

NEO-FFI (Costa & McCrae, 1992) was employed to quickly assess the Big Five personality traits - neuroticism (N), extraversion (E), openness (O), agreeableness (A), and conscientiousness (C). Each scale consists of 12 items, resulting in 60 items in total.

## Results

### Experimental Findings

Initial estimates were precisely correct responses only in 141 (2.5%) trials. When correctness was defined more loosely to encompass deviations up to five units (e.g. on „How many African countries there are in the UN?“, all values between 48 and 58 were counted as correct responses), number of correct responses rose only to 440 (7.8%) indicating that questions used in this study were relatively difficult, as intended.

Descriptive statistics for initial and final estimates on each question, as well as results of difference tests between them and associated indices of anchoring effect sizes are all presented in Table 1. Final estimates for each quantity markedly differed from the initial ones (*p*s < .05) and these differences were highly significant (*p*s < .001) on the vast majority of questions. Equally important, all of them were in predicted direction – final estimates were higher than initial estimates for positive anchors and lower for negative anchors. As much as 55.4% of initial estimates were amended toward anchors. Besides, over-anchoring was observed far more frequently than under-anchoring, χ^2^(1) = 102.56, *p* < .001. Such pattern of results confirms the experimental robustness of the anchoring effect.

**Table 1:** Descriptive Statistics and Results of Tests of Difference Between Initial (E1) and Final (E2) Estimates for Each Question Employed in the Anchoring Experiment

Distance	Question (Correct Response)	E1		E2		Difference tests		Effect size		AE	
		*M*	*SD*	*M*	*SD*	*t*(235)	*p*	*d*	η^2^	*M*	*SD*
-80%	How many squares are there on a shogi playing board? (81)	54.45	67.27	34.43	27.95	-5.54	< .001	-0.36	.12	-33.43	33.45
	How many castles are there in Vojvodina? (67)	5.84	11.04	3.71	7.61	-5.20	< .001	-0.34	.10	-24.14	29.49
	How many gold medals did China win in 2008 Summer Olympics in Beijing? (51)	15.03	24.49	11.17	21.16	-6.17	< .001	-0.40	.14	-20.96	28.12
-60%	How many chromosomes do penguins have? (80)	160.42	878.80	113.97	710.69	-2.08	.039	-0.14	.02	-24.45	26.48
	How many civil airports are there in Germany? (65)	90.81	59.53	68.11	50.23	-14.17	< .001	-0.93	.46	-25.00	19.94
	How many times was the NIN award for the best novel been awarded? (58)	21.49	46.07	13.95	23.06	-4.35	< .001	-0.28	.08	-24.67	24.55
-40%	How many African countries are there in the United Nations? (53)	8.84	11.07	6.95	8.22	-7.45	< .001	-0.48	.19	-16.13	17.38
	How long does Mercury revolution around the sun take? (88)	230.64	526.29	182.92	416.89	-5.75	< .001	-0.94	.12	-17.65	18.28
	How tall is Saint Sava Temple in Belgrade (in meters)? (82)	117.42	238.92	97.30	213.47	-5.87	< .001	-0.38	.13	-17.06	16.03
-20%	What is the length of an Olympic size swimming pool (in meters)? (50)	100.10	253.23	93.33	214.80	-2.27	.024	-0.15	.02	-4.45	8.08
	What is the duration of hockey game in minutes? (60)	53.59	25.57	49.53	24.70	-8.77	< .001	-0.57	.25	-5.80	8.88
	How long did Vuk Karadžić live? (76)	92.73	311.86	82.78	248.92	-2.42	.016	-0.16	.02	-7.33	7.93
+20%	How old is Belgrade zoo? (75)	63.19	43.07	69.97	45.40	14.50	< .001	0.94	.47	11.49	9.58
	How many keys are there on a concert grand piano? (88)	57.53	31.84	64.26	35.27	15.31	< .001	1.00	.45	12.59	9.28
	What is the hottest temperature ever recorded in the world? (58)	111.46	298.69	121.45	312.95	5.12	< .001	0.33	.10	8.60	9.16
+40%	What is the length of standard playing cards in millimeters? (88)	154.54	404.80	187.30	550.02	3.29	.001	0.21	.04	14.32	17.88
	How many novels did Agatha Christie write? (82)	23.60	28.05	28.69	33.46	9.47	< .001	0.62	.28	23.85	18.66
	How many Academy Awards for best picture was given to date? (84)	82.93	142.03	103.92	181.41	6.84	<.001	0.45	.17	25.87	18.05
+60%	What is the latitude of Reykjavik? (64)	79.90	175.18	109.94	267.42	4.67	< .001	0.30	.09	35.03	27.89
	What are 70 yards in meters? (64)	1141.50	2315.80	1227.90	2363.60	4.56	< .001	0.30	.08	25.21	28.01
	What is the atomic number of mercury? (80)	32.54	45.93	41.99	55.23	8.72	< .001	0.57	.25	34.04	28.19
+80%	How many days are there in each season of the Discordian Calendar? (73)	132.53	647.48	159.21	651.38	7.93	< .001	0.52	.21	34.62	36.66
	What is the distance between Bratislava and Vienna in kilometers? (64)	342.04	623.12	457.51	717.57	8.44	< .001	0.55	.23	41.38	34.49
	How many calories are there in a 100 grams of apple? (53)	80.84	172.01	99.70	243.37	3.09	.002	0.20	.04	24.70	33.54

*Note.* *d* = Cohen’s d. η^2^ = eta-square. *AE* = a measure of the anchoring effect.

Anchors accounted for between 2% and 50% of estimates’ variance across questions, as indicated by η^2^ values, while absolute values of Cohen’s *d* ranged from 0.14 to 1.00. On average, magnitude of anchoring effect was medium and somewhat higher for positive anchors (*d*_ANC+_ = 0.50, η_ANC+_^2^ = .20) compared to negative ones (*d*_ANC-_ = -0.42, η_ANC-_^2^ = .14).

While standard indices of effect size are directly conditioned by the response variability across participants, AE measures relate initial to final estimates for each participant and hence are not dependent on differences between participants. As it is presented in penultimate column of Table 1, final estimates were on average from 9% to 41% higher than initial estimates for positive anchors, i.e. from 4.5% to 33.4% lower for negative anchors.

**Figure 1:**
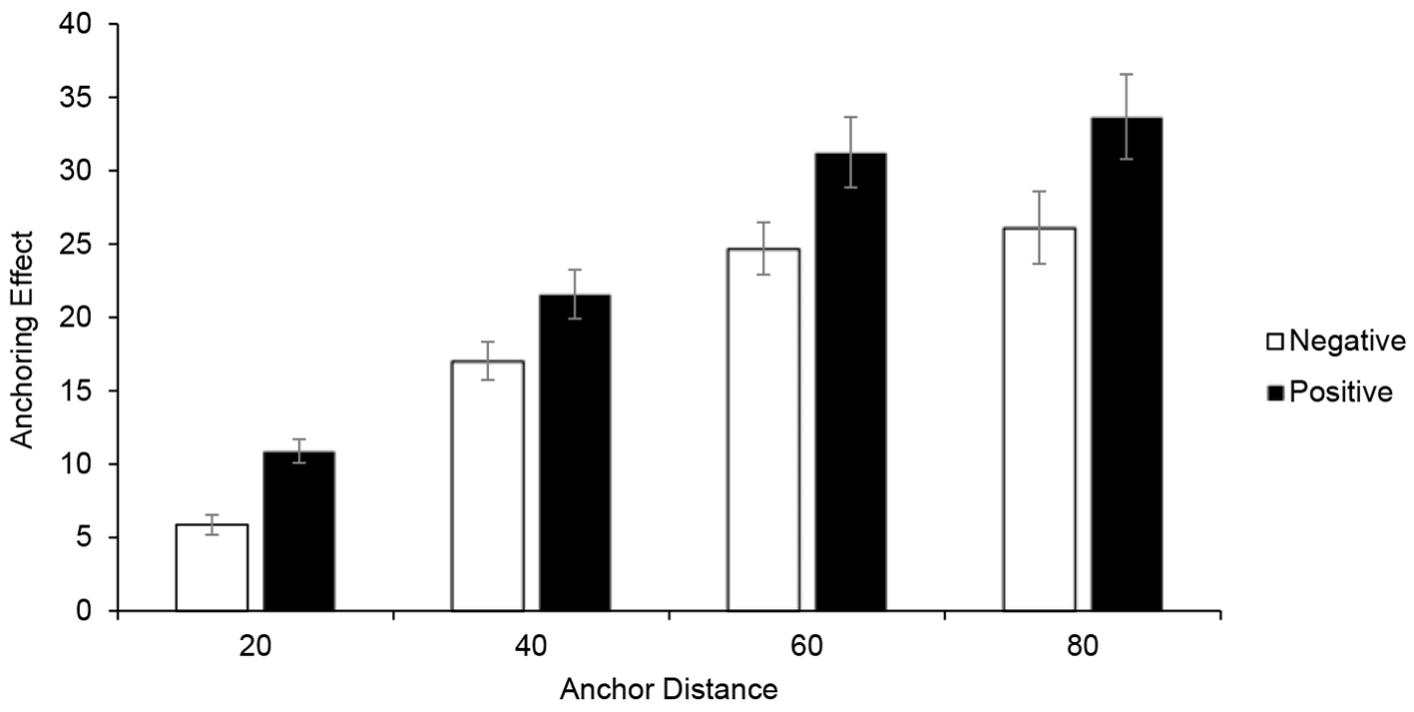
Mean anchoring effect and 95% CI error bars for each experimental condition.

To enable their comparison and further aggregation, AE values were absolutized for negative anchors. For each of eight experimental conditions, anchoring effect measures were calculated as average AE score on three belonging questions (see Figure 1).

Overall, participants amended their final estimates by 21.4 percent, 95% CI [20.3, 22.5], of initial estimates’ value. Results of two-way analysis of variance for repeated measures revealed that degree of anchoring effect strongly depended on both anchor direction, *F*(1,235) = 76.53, *p* < .001, η^2^ = .25, and anchor distance, *F*(3,705) = 265.67, *p* < .001, η^2^ = .53. Main effects were not qualified by the higher order interaction, *F*(3,705) = 1.47, *p* = .22.

Negatively directed anchors had a significantly lower impact (M_ANC-_ = 18.44, 95% CI [17.36, 19.52]) in comparison to the positive anchors (M_ANC-_ = 24.46, 95% CI [23.01, 25.91]), and this was the case for each anchor distance (*p*s < .001). The large effect of anchor’s direction is probably due to the fact that lower boundary of possible values on each question was (near to) zero.

Anchoring effect measures were even more strongly influenced by relative anchor distance. There was general increase of anchoring effect for the first three anchor distance levels. An almost linear relationship was observed with AE measures on the approximately halfway between the initial estimates and the anchors. However, increase leveled off between 60% and 80% distances, for both positive and negative anchors. Differences between mean AEs for last two distances were not significant (*p*s > .10)^iii^. This pattern of results indicates that further increasing of anchor distance would probably not be followed by significant increase of anchoring effect size.

### Susceptibility to Anchoring Effect

Considerable variability of AE measures was observed across participants on each question (see the last column of Table 1). Moreover, participants who were more susceptible to anchoring effect on one item were also more prone to amend their estimates toward anchors on other items. Internal consistency of individual differences was acceptable (α = .71). The average correlation between AE measures for 24 items was relatively small (*r* = .11), but it was notably higher after aggregation for eight experimental conditions (*r* = .27). The first principle component accounted for 36.7% of their total variance (λ = 2.94). It was highly loaded by each AE score (*r*s ranged from .46 to .73) and approximately normally distributed (KS *Z* = 0.54, *p* = .59).

### Predictors of Susceptibility to Anchoring Effect

Cognitive and personality measures were collected with the aim to explore their capacity for predicting individual differences in anchoring effect. Results presented in Table 2 suggest fair levels of reliability for all of the measures (αs ≥ .70), except for CRT (α = .40). Latter is not surprising considering that CRT consisted only of three items. Also, performance on CRT was very poor. As much as 199 (84.3%) participants failed to give at least one correct answer. Hence, a high reflective group of subjects in this study consisted of only 37 participants who scored at minimum one point on CRT.

**Table 2:** Descriptive Statistics for Predictors and Results of Multiple Regression Analysis

Predictors	Descriptive Statistics				Regression Analysis		
	*M*	*SD*	*n*	α	*r*	β	*p*
Cognitive Measures							
Raven’s Matrices (RM)	12.54	3.14	18	.79	.03	.02	.81
Vocabulary	20.82	6.49	56	.73	-.04	-.09	.17
Cognitive Reflection Test (CRT)	0.20	0.50	3	.40	.00	-.04	.70
Personality Measures							
Openness	3.26	0.53	12	.72	.24	.26	< .001
Conscientiousness	3.71	0.57	12	.84	.13	.13	.07
Extraversion	3.41	0.56	12	.78	-.01	-.08	.25
Agreeableness	3.41	0.51	12	.70	.08	.03	.67
Neuroticism	2.68	0.70	12	.84	.00	.06	.43

A measure of overall susceptibility to anchoring effect was regressed on the set of potential predictors^iv^ and the results are displayed in the last three columns of Table 2. Zero-order correlations were relatively small, and only the trait of openness correlated significantly with anchoring effect (*r* = .24, *p* < .001). Besides, openness was the only measure that significantly contributed to the prediction model (β = 0.27, *p* < .001). In total, relatively small portion of anchoring effect’s variance was accounted for by predictors (*F*(8,227) = 2.65, *p* = .008, *R*^2^ = 5.3%).

Cognitive measures showed no direct correlation with anchoring. However, it was hypothesized that processes captured by AE measures may differ with respect to the degree of subjects’ reflectivity, i.e. that cognitive reflection might moderate the relationship between cognitive abilities and anchoring effect. Results confirmed this expectation. Standardized interaction term, entered in multiple regression analysis along with standardized CRT and RM scores, was highly significant (β = -.24, *p* = .004). For the group of participants who performed poorly on CRT (N_CRT-_ = 199), the correlation between RM scores and anchoring effect measures was not significant (*r* = .11, *p* = .13), while for the group of participants that had at least one correct CRT answer (N_CRT+_ = 37), RM performance significantly correlated with anchoring (*r* = -.51, *p* = .001)^v^. Furthermore, results of separate multiple regression analyses for the two groups, presented in Table 3, indicate stronger effect of predictor set in high reflective group (*R*^2^ = 32.0%) in comparison to low reflective group (*R*^2^ = 5.3%), even after controlling for difference between size of groups (Fischer’s *z* = 2.21, *p* = .027).

**Table 3:** Results of Regression Analyses for Two Cognitive Reflection Groups

Predictor	Low CRT^a^		High CRT^b^	
	β	*p*	β	*p*
Raven’s Matrices	.08	.29	-.49	.003
Vocabulary	-.05	.50	-.29	.049
Openness	.26	.001	.19	.26
Conscientiousness	.10	.23	.18	.28
Extraversion	-.06	.44	-.19	.28
Agreeableness	.01	.84	.18	.28
Neuroticism	.08	.34	-.07	.72

^a^*F*(7,191) = 2.592, *p* = .014, *R*^2^ (adjusted) = 5.33%. ^b^*F*(7,29) = 3.422, *p* = .009, *R*^2^ (adjusted) = 32.01%.

## Discussion

Anchoring effect is a well-documented phenomenon. Yet, studies that examine covariates of susceptibility to this cognitive bias are relatively recent and with mixed findings (Bergman et al., 2010; Caputo, 2014; Eroglu & Croxton, 2010; Furnham et al., 2012; Jasper & Christman, 2005; Jasper & Ortner, 2014; McElroy & Dowd, 2007; Oechssler et al., 2009; Stanovich & West, 2008; Welsh et al., 2014). Relative inconsistency in reported results could be partly due to questionable psychometric properties of used instruments, but also due to the absence of the uniform procedure for reliable measurement of individual differences in susceptibility to anchoring effect. In respect to latter, at least two approaches can be distinguished. First, predictors of anchoring were examined by using interaction test in 2 x 2 ANOVA with anchor condition (high/low) and dichotomized psychometric construct of interest as between-subject factors. In such way, McElroy and Dowd (2007) showed that participants who were high on openness to experience provided higher estimates for high anchors and lower estimates for low anchors in comparison to participants who were low on openness. A similar procedure was used in several subsequent studies (Furnham et al., 2012; Oechssler et al., 2009; Stanovich & West, 2008). Drawbacks of this approach lie in the inevitable arbitrariness of dichotomization criteria and practical inability to simultaneously examine effects of several predictors. As an alternative solution, Welsh et al. (2014) proposed a multi-item measure of anchoring expressed as a correlation between experimentally provided anchors and participant’s numerical estimates across the number of trials on a specially constructed card game. In such way, several predictors could be simultaneously examined. However, authors missed to examine reliability of these measures, and the question of generalizability of findings beyond gambling setting can also be raised.

The present study was designed with an intention to overcome some shortcomings of the previous studies. Introduction of pretest session, in which participants stated their anchor-free estimates before appliance of standard paradigm, ensured that anchor direction and relative extremity were same for all participants. This also allowed direct comparability of the anchor distance and the anchoring effect measures. An additional benefit of applied within-subject procedure lies in the opportunity to collect measures of the anchoring effect for each participant on each question and to evaluate psychometric properties, which was mostly ignored in previous research (for the exception, see Jasper & Ortner, 2014). Finally, proposed procedure opens the possibility for multilevel analyses aimed to explore whether effects of experimental factors are moderated by individual characteristics of participants^vi^.

The average size of the anchoring effect in the present study was medium. Participants amended an initial estimate of uncertain quantity toward anchor in more than half cases, on average for slightly more than one-fifth of its value. In that sense, an experimental reliability of the anchoring effect was yet again confirmed, this time by using within-subject design. It seems plausible to suppose that anchoring effect would be even stronger if participants were not previously asked to express their anchor-free estimates, i.e. that reported results can be seen as conservative indications of the anchoring effect size.

Anchors showed asymmetrical effect. Positively directed anchors produced larger effects in comparison to equally distanced negative anchors, which is in line with previous findings (Hardt & Pohl, 2003; Jacowitz & Kahneman, 1995; Jasper & Christman, 2005). This was probably due to fact that the degree of estimate change for negative anchors was both theoretically and psychologically limited by a fixed lower boundary of possible responses. Unlike that, effects of positive anchors were not limited in this way, since upper boundaries of reasonable values on the most of questions were not fixed. Besides, in both directions, more distanced anchors led to a larger anchoring effect, but only to some point. For the most extreme anchors, no significant difference in effect was observed in comparison to the nearest anchors. This indicates that maximum of the anchoring effect was registered and that more distant anchors would yield same (Mussweiler & Strack, 2001) if not weaker (Wegener et al., 2001) effect.

Considering the correlational aspects, results indicate fair internal consistency of the anchoring effect measures, thus preventing alternative interpretations of negligible correlations. Anchoring effect was directly associated only with the trait of openness. As suggested by McElroy and Dowd (2007), this can be viewed as a consequence of enhanced sensitivity to external information which is a common characteristic of two phenomena. In other words, participants prone to take into account alternative points of view in general also show increased readiness to amend their answers toward externally suggested solutions on estimation tasks. Other personality traits were not correlated with anchoring, although some previous studies suggested that possibility (e.g. Caputo, 2014; Eroglu & Croxton, 2010).

Findings on the relations between cognitive variables and AE measures are of particular theoretical importance since they could shed some light on the discussion about psychological mechanisms that underlie anchoring effects. Kahneman (2011) put previously emerged distinction between two competing theoretical accounts of the anchoring effect into the context of dual-process theories of human reasoning. An adjustment was explicitly described as deliberate Type 2 process which is typically carrying out in multiple steps. After estimating if the correct value is higher or lower than the presented anchor, people adjust from the anchor by generating an initial value. Afterwards, people evaluate if this value is a reasonable answer (in which case they terminate adjustment and provide an estimate), or it requires additional modification (in which case they readjust estimate further away from the anchor value). Anchoring effect is hypothesized to arise partially because the capability to perform processes of evaluating and adjusting is limited by capacities of working memory. Since individual differences in these capacities can be approximated by intelligence tests (e.g. Evans & Stanovich, 2013; Stanovich & West, 2008), one would expect to observe a negative correlation between measures of intelligence and anchoring. In that sense, finding that AE measures were not directly related to cognitive measures, which is in line with previously reported results (Bergman et al., 2010; Furnham et al., 2012; Jasper & Ortner, 2014; Oechssler et al., 2009; Stanovich & West, 2008; Welsh et al., 2014), could imply that adjustment, as effortful cognitive process is not involved in the emergence of the anchoring effect at all. Nevertheless, a relation between intelligence and anchoring depended on cognitive reflection. In other words, readiness to involve in more demanding Type 2 processes moderated the relation between cognitive capacities and susceptibility to anchoring effect. For the vast majority of participants, who were prone to mentally economize by hinging on intuitive Type 1 processes, differences in intelligence indeed were not related to anchoring. It can be hypothesized that final estimates of these subjects have not resulted from serial adjustments but were outcomes of Type 1 processing, i.e. relatively automatic activation of information consistent with the presented anchor. Observed variability among these subjects might be due to variations in the dispositional sensitivity to external information, or due to individual differences in distortions of scale on which answers are provided (e.g. Frederick & Mochon, 2012), but unlikely due to capacities to repeatedly reconsider readjusted anchor as a possible answer to given question. However, for more reflective subjects, negative correlation between intelligence and anchoring was observed – participants with higher capacities to carry out Type 2 processes adjusted further away from anchors. In other words, differences among more reflective subjects, who were prone to engage in processes of reevaluation and readjustment before stating their final estimates, were at least to some extent due to their capacities to perform these processes. This indicates that (insufficient) adjustment, at least for some subjects, plays role in the emergence of the anchoring effect.

This finding, though, should be taken with caution, considering the low reliability of three-item CRT and relatively small size of high reflective subsample. Future replications are needed^vii^. While more direct ones would seek to perform similarly designed study by using psychometrically improved instruments, such as seven-item CRT (Toplak, West, & Stanovich, 2014), others could examine whether comparable results would be obtained if cognitive reflection is measured in other ways, for example by using typical performance measures, such as need for cognition or actively open-minded thinking^viii^, as suggested by Stanovich (2009). Finally, studies that combine experimental and differential perspective could be also applied with the same purpose. For example, study could be organized with aim to explore if subjects with larger cognitive capacities benefit more from interventions previously shown to reduce anchoring effect, such as consider-the-opposite strategy (Adame, 2016; Mussweiler, Strack, & Pfeiffer, 2000), forewarnings (Epley & Gilovich, 2005, Study 2), or accuracy motivation (Epley & Gilovich, 2005, Study 1; Simmons et al., 2010).

The present study explored some of the potential benefits of novel within-subject procedure which allows simultaneous manipulation of experimental factors of anchor’s distance and direction and reliable measurement of individual differences in susceptibility to anchoring effect. Although first results are promising, future studies are needed, especially those driven by theoretical expectations aimed to examine cognitive processes involved in anchoring effect.

## Appendix

**Table A1:** Distribution of Missing and Trimmed Data in the Anchoring Experiment

Anchor	Target quantity (correct response)	Zero denominators	Over-anchoring	Under-anchoring
-80%	Squares on a shogi playing board (81)	1	5	16
	Castles in Vojvodina (67)	23	2	6
	Gold medals won by China in 2008 Summer Olympics (51)	3	0	33
-60%	Chromosomes in penguins (80)	5	6	9
	Civil airports in Germany (65)	2	1	11
	Awarded NIN prizes for best novel (58)	0	5	35
-40%	African countries in the United Nations (53)	20	6	14
	Mercury revolution around the sun in days (88)	3	13	22
	Heights of Saint Sava Temple in meters (82)	0	6	7
-20%	Length of an Olympic size swimming pool in meters (50)	0	15	15
	Duration of the hockey game in minutes (60)	0	12	8
	Years lived by Vuk Karadžić (76)	0	3	10
+20%	Belgrade zoo in years (75)	0	36	16
	Concert grand piano keys (88)	0	68	12
	Hottest temperature recorded in the world (58)	0	36	13
+40%	Length of standard playing cards in millimeters (88)	0	28	9
	Novels that Agatha Christie wrote (82)	5	80	7
	Academy Awards for best picture (84)	0	75	17
+60%	Latitude of Reykjavik (64)	2	56	12
	Seventy yards in meters (64)	5	34	18
	Atomic number of mercury (80)	5	93	4
+80%	Days in each season of the Discordian Calendar (73)	5	30	13
	Distance between Bratislava and Vienna in kilometers (64)	1	25	8
	Calories in a 100 g apple (53)	12	17	19
Total		92 (1.63%)	652 (11.56%)	334 (5.92%)

**Table A2:** Correlations Among Cognitive Variables and Personality Traits

Variable	1	2	3	4	5	6	7
Cognitive Measures							
1. Raven’s Matrices (RM)	–						
2. Vocabulary	.30**	–					
3. Cognitive Reflection Test (CRT)	.23**	.09	–				
Personality Measures							
4. Openness	.23**	.16*	.18*	–			
5. Conscientiousness	-.11	-.06	-.05	.12	–		
6. Extraversion	-.09	-.20*	.08	.20**	.22**	–	
7. Agreeableness	.09	.08	.00	.17**	.17**	-.02	–
8. Neuroticism	-.11	.03	-.03	-.12	-.40**	-.37**	-.19**
